# Improving Temporal Stability and Accuracy for Endoscopic Video Tissue Classification Using Recurrent Neural Networks

**DOI:** 10.3390/s20154133

**Published:** 2020-07-24

**Authors:** Tim Boers, Joost van der Putten, Maarten Struyvenberg, Kiki Fockens, Jelmer Jukema, Erik Schoon, Fons van der Sommen, Jacques Bergman, Peter de With

**Affiliations:** 1 Department of Electrical Engineering, Eindhoven University of Technology, Groene Loper 3, 5612 AE Eindhoven, The Netherlands; J.A.v.d.Putten@tue.nl (J.v.d.P.); fvdsommen@tue.nl (F.v.d.S.); P.H.N.de.With@tue.nl (P.d.W.); 2 Amsterdam University Medical Center, Meibergdreef 9, 1105 AZ Amsterdam, The Netherlands; m.r.struyvenberg@amsterdamumc.nl (M.S.); k.n.fockens@amsterdamumc.nl (K.F.); j.b.jukema@amsterdamumc.nl (J.J.); j.j.bergman@amsterdamumc.nl (J.B.); 3 Catharina Hospital, Michelangelolaan 2, 5623 EJ Eindhoven, The Netherlands; erik.schoon@catharinaziekenhuis.nl

**Keywords:** Barrett neoplasia, tissue detection, recurrent neural networks, upper GI tract

## Abstract

Early Barrett’s neoplasia are often missed due to subtle visual features and inexperience of the non-expert endoscopist with such lesions. While promising results have been reported on the automated detection of this type of early cancer in still endoscopic images, video-based detection using the temporal domain is still open. The temporally stable nature of video data in endoscopic examinations enables to develop a framework that can diagnose the imaged tissue class over time, thereby yielding a more robust and improved model for spatial predictions. We show that the introduction of Recurrent Neural Network nodes offers a more stable and accurate model for tissue classification, compared to classification on individual images. We have developed a customized Resnet18 feature extractor with four types of classifiers: Fully Connected (FC), Fully Connected with an averaging filter (FC Avg (n = 5)), Long Short Term Memory (LSTM) and a Gated Recurrent Unit (GRU). Experimental results are based on 82 pullback videos of the esophagus with 46 high-grade dysplasia patients. Our results demonstrate that the LSTM classifier outperforms the FC, FC Avg (n = 5) and GRU classifier with an average accuracy of 85.9% compared to 82.2%, 83.0% and 85.6%, respectively. The benefit of our novel implementation for endoscopic tissue classification is the inclusion of spatio-temporal information for improved and robust decision making, and it is the first step towards full temporal learning of esophageal cancer detection in endoscopic video.

## 1. Introduction

Artificial intelligence (AI) systems exceeding expert performance have shortcomings when they are applied on data outside their training domains. At present, such AI systems lack a form of context awareness, which allows the model to reject data outside its learned feature space. Since medical examinations often include an extensive range of anatomical checks, there is a risk that AI-based automated lesion detectors will be applied outside the target domain. Potentially, when inexperienced clinicians are relying on the algorithm, this might lead to higher false positives and false negatives and thereby to malignancies in the diagnosis, which is to the detriment of patients. Assistive tools for automatic lesion detection should therefore be designed for robustness and accuracy with the standard clinical practice in mind.

In the field on gastroenterology, a Computer-Aided Detection (CAD) system has been developed for Barrett’s neoplasia detection in white light endoscopic still images [1], achieving expert performance. Yet, this algorithm is restricted and validated on the visual features of a Barrett’s esophagus (BE). In clinical practice, it is common to fully assess the esophagus from stomach to the healthy squamous region. Therefore, the current model should only be restricted to the analysis of the Barrett’s region of the esophagus.

In order to facilitate the continuous analysis of the video signal during the full clinical protocol, a vast pool of new relevant and irrelevant features needs to be taken into account. For example, optical tissue deformation, which can be estimated through consecutive frames, is an inherent cell marker for testing malignant morphological changes, according to Guck et al. [2]. In contrast, when ambiguous frames are introduced, the model could become unstable according to Van der Putten et al. [3]. In order to deal with such ambiguity, the model should consider the context of prior frames for robust and reliable decision making. The consecutive frames in an endoscopy procedure do not differ substantially, and therefore information prior to an ambiguous frame can be exploited to make an accurate prediction. Such sequential models could be used to improve position tracking in during an endoscopy procedure. Accordingly, since Esophagus Adenocarcinoma (EAC) only occurs in a particular segment of the esophagus (i.e., in BE), frames that are captured outside this segment could be disregarded by an EAC detection algorithm, leading to a reduction in false alarms and an increased user confidence in the CAD system.

Practically, different approaches and algorithms have been applied on time-series data, including independent frame analysis, averaging over the temporal domain and hidden Markov models [4,5,6,7,8]. However, the absence of long-term memory in these models hampers the exploitation of long-distance interactions and correlations, which make the corresponding algorithms not suitable for learning long-distance dependencies typically found in clinical data. Since the employed, existing image-based classification networks are trained on still images in overview, the response on unseen non-informative frames is unknown. This implies that algorithms trained only on still images do not perform well on video signals without algorithm modifications. [9]

Recurrent Neural Networks (RNNs) can be used to provide a temporal flow of information. These networks have been widely used to learn the processing of sequential video data and are capable of dealing with long-term dependencies. In this type of artificial neural network, connections are formed between units and a directed cycle. This cycle creates an internal state of the network which allows it to exhibit and model dynamic temporal behavior without computation-intensive 3D convolutional layers. Recently, Yao et al. [10] demonstrated a state-of-the-art method for action recognition, which imposes Gated Recurrent Units (GRUs) on the deep spatiotemporal information extracted by a convolutional network. Furthermore, Yue et al. [11] and Donahue et al. [12] have successfully demonstrated the ability of RNNs to recognize activity, based on a stack of input frames. A similar approach could be followed for the classification of tissue in videos, thereby potentially leading to a more temporally stable algorithm, since it is able to exploit information existing in the temporal domain.

The literature describes a variety of methods to analyze video for classification tasks in endoscopy. The most basic form for video analysis describes a single frame based analysis for classification [13,14,15]. Other recent work on video analysis in endoscopy focuses on a frame-based analysis approach with additional post processing to yield some form of temporal cohesion. Byrne et al. [16] describe a frame-based feature extractor, which interpolates a confidence score between consecutive frames, in order to make a more confident prediction for colorectal polyp detection. De Groof et al. [9] implement a voting system for multiple frames on multiple levels. Yu et al. [17] describe a 3D convolutional model, in order to capture inter-frame correlations. Yet, 3D convolutions fail to capture long-term information. Harada et al. [18] propose an unsupervised learning method, which clusters frame-based predictions, in order to improve temporal stability in tissue classification. Yet, a clustering approach is not able to capture the consecutive or inter-frame correlation between frames. Frameworks that do actively learn spatiotemporal information with the implementation of RNNs are described by, Owais et al. [19] and Ghatwary et al. [20]. They demonstrate that the implementation of RNNs yield superior classification accuracies in endoscopic videos, but no quantitative results are reported on the stability of the employed models.

In this paper, we address the ambiguity in the classification of tissue in the upper gastrointestinal tract by introducing RNNs, as a first exploratory study to obtain a more robust system for endoscopic lesion detection. Our system is generally applicable for CAD systems in the gastrointestinal tract and can potentially serve as a pre-processing step that reduces the amount of false alarms for a wide range of endoscopic CAD systems. We hypothesize that by extending Resnet18 with RNNs, or more specifically, by employing Long Short Term Memory (LSTM) or Gated Recurrent Unit (GRU) as concepts, the model is able to actively learn and memorize information seen in earlier frames to make a more accurate prediction about the tissue class compared to networks without temporal processing.

Our contributions are therefore as follows. First, our work demonstrates that including temporal information in endoscopic video analysis leads to an improved classification performance. Second, We show that exploiting the concepts of LSTM and GRU outperform the conventional Fully Connected (FC) networks. Third, the proposed approach offers a higher stability and robustness in classification performance, so that it paves the way for applying automated detection during the complete clinical endoscopic procedure.

## 2. Materials and Methods

### 2.1. Ethics and Information Governance

This work and the involved local collection of data on implied consent, received national Research Ethics (IRB) Committee approval from the Amsterdam UMC (No. NTR7072). De-identifcation was performed in line with the General Data Protection Regulation (EU) 2016/679.

### 2.2. Datasets and Clinical Taxonomy

To train and evaluate the classification performance of our networks, we collected a dataset consisting of 82 endoscopic pullback videos from 75 patients, which were recorded prospectively in the Amsterdam UMC. Written informed consent was obtained from all participants. In total, 46 out of the 82 videos were derived from patients who were diagnosed with high-grade dysplasia. These videos were captured using White Light Endoscopy in full high-definition format (1280 × 1024 pixels) with the ELUXEO 7000 endosco py system (FUJIFILM, Tokyo, Japan). During the recording of a pullback video, the endoscope is slowly pulled from the stomach up to the healthy squamous esophagus tissue in one smooth sequential movement.

**Figure 1:**
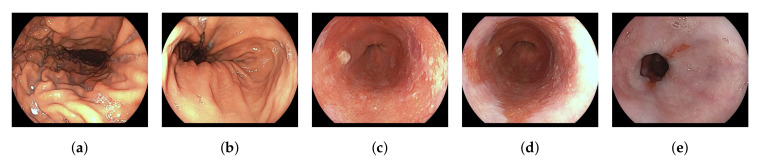
Visual examples for each tissue label to be classified by the model sourced from pullback videos. These pullback videos start recording from the (**a**) Stomach and stops, while pulling the endoscope with a constant speed, at the (**e**) Squamous area. (**a**) Stomach; (**b**) Transition-zone Z-line; (**c**) Barrett; (**d**) Transition-zone Squamous; (**e**) Squamous.

In our processing, we have sampled with 5 frames per second at a resolution of 320×256 pixels. Each resulting frame is manually labeled by one out of three experienced clinicians with respect to tissue class and informativeness. The five tissue classes are ‘stomach’, ‘transition-zone Z-line’, ‘Barrett’, ‘transition-zone squamous’ and ‘squamous’, see Figure 1. Frames are labeled as non-informative if they have: out-of-focus degradation, visible esophagus contractions, video motion blur, broad visibility of bubbles, or excessive contrast from lighting. For the training, we have only selected sequences in which the last frame is labeled informative. From the total dataset consisting of 20,663 frames, 19,931 are labeled as informative by a team of three clinical research fellows.

### 2.3. Network Architectures and Training Protocol

Our network architecture is split in two principal stages: a feature extractor followed by a classification network. The feature extraction is based on a modified ResNet18 network, containing four fully convolutional blocks. This model is applied in many fields for image classification, and should therefore allow for an easier comparison to other literature. Different classifiers are evaluated in our experiments. Since we only want to measure the effect of applying RNNs on the model robustness, we keep the classifiers as simplistic as possible. The following classifier configurations are tested: (1) two Fully Connected layers (FC) with a Rectified Linear Unit function in between, (2) a two-layer LSTM directly followed by a fully connected layer, and (3) a two-layer GRU directly followed by a fully connected layer. Both LSTM and GRU classifiers are assembled with two recurrent layers, since according to Graves et al. [21] depth is needed to increase the receptive field of an RNN. Both LSTM and GRU are trained with a hidden state size of 128 parameters. Since relevant literature also describes methods to exploit temporal information based on single image features, we also apply a simplistic averaging filter over 5 frames to the FC classifier in order to smooth the output over the temporal domain. This classifier is denoted as FC Avg (n = 5).

**Figure 2:**
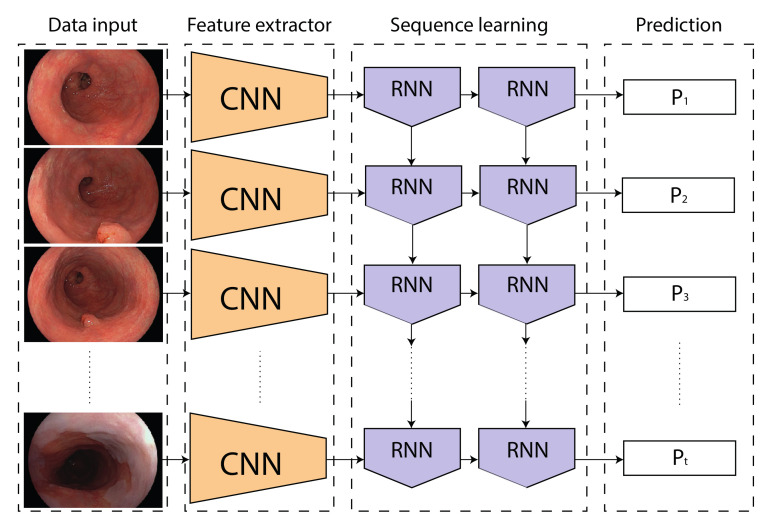
Data flow through the proposed model during training. Recurrent Neural Network (RNN) represents either Long Short-Term Memory (LSTM) or Gated Recurrent Unit (GRU) nodes.

We introduce random under-sampling for training set stratification, by taking into account the asymmetry of the dataset. Doing so, each video frame is assigned a weight, so that the probability of sampling each class and case, is equal. Per iteration, we randomly sample 512 sequences consisting out of only one or 10 consecutive frames, to facilitate individual or temporal classification, respectively. The flow of data through our proposed model for temporal classification is illustrated in Figure 2.

The network is trained using Adam optimization with an initial learning rate of $10^{-4}$. A cyclic cosine learning-rate scheduler is used to further control the learning rate. A cross-entropy loss is implemented to converge the neural network. For data augmentation, random-affine transformations are applied during training with rotations up to 5 degrees, translation and cropping of 2.5% of the image length and shearing up to 5 degrees. These parameters are kept constant per sequence.

## 3. Experiments

This section first describes the metrics for measuring the performance of our method, and then follows with the statistical analyses of the various classes and configurations to obtain a broad set of experimental results.

### 3.1. Metrics

The reported performance of the model is measured with two different metrics, i.e., stability and accuracy. The stability of the network is determined by the average amount of times the network switches from predicted label per video. Thus, if at time $t_{n}$, the model predicts label *a*, and at $t_{n+1}$ the model predicts label *b*, the predicted labels switch from *a* to *b*. This change is counted as one domain switch. Since our dataset contains 5 labels, and each label should be passed only once, a perfect score would be 4 label switches. The accuracy is measured as the accuracy score averaged per patient, as shown in Equation (1). The label accuracy is averaged per patient, in order to normalize for a variable video length, and specified by

(1) $$\mathrm{Mean}\;\mathrm{label}\;\mathrm{Accuracy}=\frac{1}{N_{p}}\sum_{i=1}^{N_{p}}\mathrm{Acc}\left(L_{i}\right),$$

where, $L_{i}$ denotes the label of event *i* and Acc(.) is the accuracy function. In Equations (1) and (2), $T_{P}$ is true positive, $N_{v}$ is the number of frames, and $N_{p}$ is the number of patient cases. True positives are defined as in Table 1 and specified by

(2) $$\mathrm{Label}\;\mathrm{Acc}(L)=\frac{1}{N_{v}}\sum_{j=1}^{N_{v}}T_{P}{}_{j}.$$

The explanation of Table 1 is as follows. For all rows where double or triple labels are indicated, we mean that if the predicted label has a ground truth in one of the two/three labels, then the prediction label is considered correct. For example, if Barrett’s tissue is predicted and deemed correct, this means that the ground-truth labels from the clinician annotation may be ‘Transition Z-line’, ‘Barrett’ or ‘Transition Squamous’.

This mapping of the annotations labels from the ground truth is chosen to compensate for the ambiguity of the transition zones, since the transition zones adjacent to a distinct class are now mapped onto that class. Consequently, this metric will better separate non-metaplastic and metaplastic tissue. This is important because a metaplastic area is the region of interest to examine for Barrett’s neoplasia.

### 3.2. Statistical Analysis

The network performance is evaluated using a fivefold cross-validation. The dataset is divided into five equally-sized folds, such that in each fold each individual patient is represented once. The performance reported in this paper is the average accuracy over all folds and patients. To substantiate the statistical significance, we will also perform a Wilcoxon signed-rank test [22]. This is a nonparametric test applied to matched-pair data, which tries to find a distribution centered around zero based on differences.

## 4. Results

**Figure 3:**
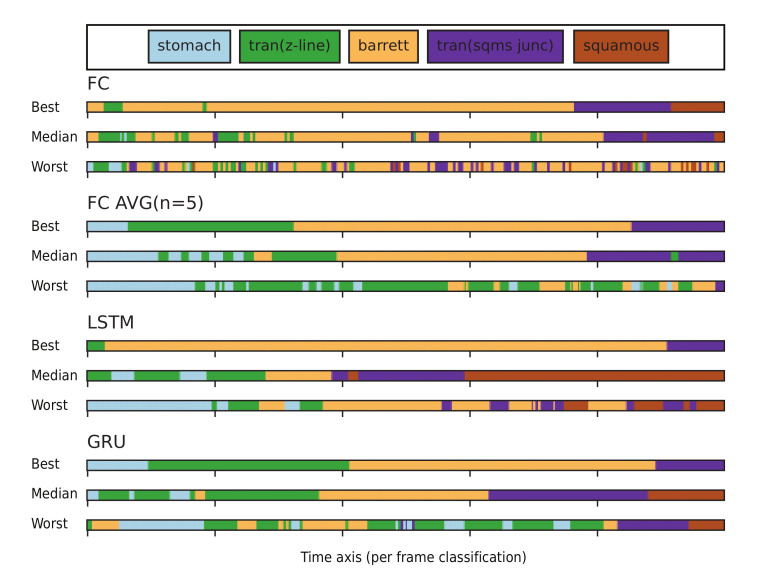
Each bar illustrates the predicted organ labels per frame over a time axis. The figure illustrates the instability of the compared networks architectures at three different performance levels, which are best, median and worst performance for each model respectively. The average domain switch for Fully Connected classifier, is 43.27, Fully Connected Averaged (n = 5) 18.49, Long Short-Term Memory classifier 10.81 and Gated Recurrent Unit classifier 11.91

The output stability is measured for four models, FC, FC Avg(n = 5), LSTM and GRU. We have found on average that the following networks switches from label: FC 43.27 (±23.83), FC Avg (n = 5) 18.48 (±9.76), LSTM 10.81 (±5.68) and GRU 11.91 (±6.33). These results demonstrate that the models implemented without RNNs, switch 2–4 more times from label within a single video. This is especially apparent in the video’s in which the model has a bad performance, see Figure 3.

**Figure 4:**
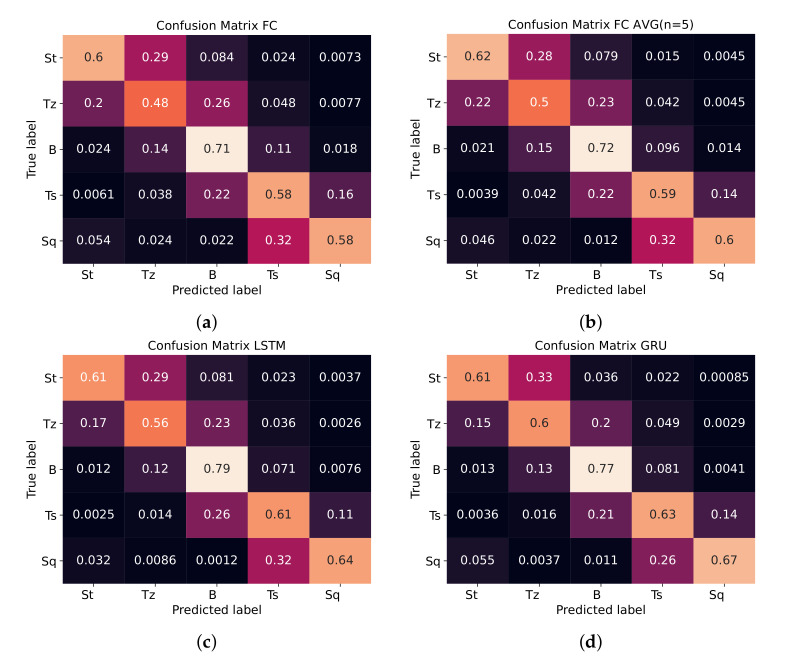
Confusion matrices display the average percentage that a True Label is predicted as a specific class. These values are normalized for the patient cases. (**a**) Fully Connected classifier, (**b**) Fully connected Averaged (n = 5), (**c**) Long Short-Term Memory classifier, and (**d**) Gated Recurrent Unit classifier.

Table 2 displays the mean accuracy of the four different models for the five different tissue classes. The results show that by averaging over five consecutive frames, a performance improvement of 0.8% is obtained. The introduction of RNNs into the classification model results yield an increase of 3.7% in overall accuracy, as seen with the accuracies of 85.9% and 85.6% for LSTM and GRU, respectively, compared to 82.2% for FC. Detailed performances per model for each class are provided in confusion matrices in Figure 4. The Wilcoxon signed-rank test has found a p<0.001 in all comparisons on accuracy of FC, LSTM and GRU classifiers, which confirms the statistical significance of the results.

An important observation is the accuracy of 98.3% on the Barrett’s segment in the esophagus. A good performance on this label is crucial, since this model will be used in an a priori tissue classification to extend the robustness for lesion detection. High sensitivities are generally preferred in this field, since a false positive will only lead to an extra biopsy, while a false negative gives a severe detriment to the patient. The demonstrated accuracy score implies that roughly 1.7% of the images are rejected due to lesion detection caused by a false negative. We consider that this is acceptable because during inference, we are able to process up to 180 frames per second for real-time video analysis. This would mean that even if some frames are rejected, the time-gap between the analyzed frames would be small, so that the Barrett area will still be fully analyzed effectively.

## 5. Discussion and Conclusions

In this work, we have explored the use of Recurrent Neural Networks (RNNs) for true temporal analysis of endoscopic video. In particular, we have evaluated two popular RNN architectures (i.e., Long Short-Term Memory (LSTM) and Gated Recurrent Unit (GRU)) for tissue classification in endoscopic videos. This is a particularly interesting application, since current CAD systems show a relatively high number of false classifications for video frames captured outside the organ of interest. Reliably detecting the organ that is currently in view can therefore lead to an increased CAD performance. We demonstrate that by exploiting temporal information in latent space, much more stable classification behavior is observed than when simple frame averaging is used. Hence, the results confirm our hypothesis that by leveraging RNNs, we can stabilize the classification output from the model. Moreover, by learning the temporal flow we have also discovered an increase in the accuracy of all tissue classes. For the application of Barrett’s cancer detection, the proposed system reliably detected the tissue of interest, i.e., the Barrett label, with an accuracy of 98.3%. These results are a proof of concept, and therefore the presented models do not yield the optimal results. In future work we will address this limitation by conducting an ablation study to find the optimal parameters.

The classification performance on the stomach and squamous tissue remains relatively poor. This discrepancy can be observed in Table 2 and is mostly caused by the definition of the label correspondence mapping in Table 1. Although the algorithm is able to approximate the tissue type, it often also guesses the neighboring tissue type. This error can be readily understood, as there is no hard defined transition on the visible border between tissue types, i.e., each view gradually transitions over time into the next one, resulting in the property that adjacent tissue areas (and labels) visually exhibit similar features (see Appendix A).

To address this transition ambiguity, a score based on the agreement between observers could be introduced. However, in our current training protocol, we only have one annotated label available per frame, originating from one of the three observers. By introducing multiple observers per frame, a score of agreement can be calculated (like simple majority voting), which can be used to train the future algorithm. Such an approach would take into account the ambiguity, and can then potentially also result into an additional score for ambiguity.

An other limitation is that the employed data is imbalanced at present. As can be seen in Table 2, the labels Stomach and Squamous are under-represented in the dataset. This imbalance is partly a reason for the poor performance on these classes. To overcome the limitation of available data, future efforts will focus on the collection of data, originating from other sources than videos alone.

In conclusion, our work has demonstrated that incorporating temporal information in endoscopic video analysis can lead to an improved classification performance. Exploiting the sequential bias present in endoscopic video (e.g., the order of the tissue types that are captured, in addition to a higher accuracy), also presents a more stable classification behavior over time. Although being directly applicable to EAC detection in BE patients, to likely enhance the CAD performance by reliably detecting the Barrett’s tissue, our approach can be generalized and easily translated to similar endoscopic video analysis tasks. Future experiments should explore such novel applications and should focus on combining the proposed pre-processing system with several succeeding, and already established classification tasks. 

## Figures and Tables

**Table 1:** Correspondence between predicted labels and ground-truth annotations of the clinicians.

Predicted Label	True Positive If Label Is:
Stomach (St)	St
Transition Z-line (Tz)	Tz, B
Barrett (B)	Tz, B, Ts
Transition squamous (Ts)	B, Ts
Squamous (Sq)	Sq

**Table 2:** Mean accuracy per tissue class, and for various architecture configurations. Scores are averaged over all patient cases. The mean label accuracy is in correspondence with the label classification from Table 1. The classifiers reported are Fully Connected classifier, Fully Connected Averaged (n = 5), Long Short-Term Memory classifier, and Gated Recurrent Unit classifier.

Label	N	Mean Accuracy (%)			
		FC	FC Avg (n = 5)	LSTM	GRU
Stomach (St)	2593	59.6	62.2	60.7	**61.3**
Tran. Z-line (Tz)	2921	74.1	72.9	79.3	**79.7**
Barrett (B)	9444	95.7	96.1	98.0	**98.3**
Tran. squamous (Ts)	4215	79.5	81.3	**87.0**	83.9
Squamous (Sq)	755	58.3	59.9	63.8	**67.4**
Overall	19,931	82.2	83.0	**85.9**	85.6

**Figure A1:**
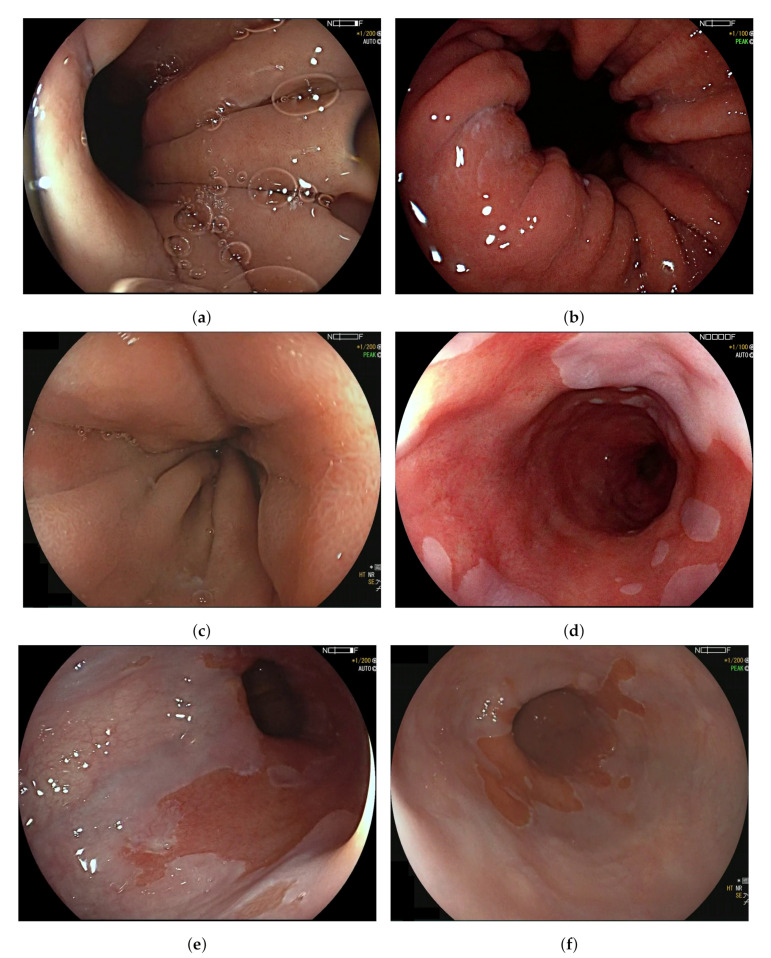
Examples of images with similar imaging features, which are lacking a hard definable border for a classification label. (**a**–**c**) contain images that represent the transition area from stomach to the Barrett’s esophagus, which all contain features, like gastric folds, and (**a**,**b**) also include a view into the stomach. (**d**–**f**) contain representations seen around the transition from Barrett’s esophagus to healthy squamous tissue, which all contain visual features from the Barrett’s esophagus and squamous tissue. (**a**) Cardia of the stomach labeled as ‘Stomach’. (**b**) Image captured after the esophageal sphincter labeled as ‘transition Z-line’. (**c**) Contracted Barrett’s esophagus labeled as ‘Barrett’. (**d**) Barrett’s esophagus with healthy squamous tissue falling into the field of view labeled as ‘Barrett’. (**e**) Transition zone between Barrett’s esophagus and healthy squamous tissue, labeled as ‘transition squamous. (**f**) Mostly squamous tissue, with Barrett’s esophagus in the background, labeled as ‘squamous’.

## Appendix A. Ambiguous Organ Classes

This section illustrates examples of ambiguous frames due to lacking a clearly defined transition between tissue classes.
